# Surveillance for pneumonic plague in the United States during an international emergency: a model for control of imported emerging diseases.

**DOI:** 10.3201/eid0201.960103

**Published:** 1996

**Authors:** C L Fritz, D T Dennis, M A Tipple, G L Campbell, C R McCance, D J Gubler

**Affiliations:** Centers for Disease Control and Prevention, Fort Collins, Colorado, USA.

## Abstract

In September 1994, in response to a reported epidemic of plague in India, the Centers for Disease Control and Prevention (CDC) enhanced surveillance in the United States for imported pneumonic plague. Plague information materials were rapidly developed and distributed to U.S. public health officials by electronic mail, facsimile, and expedited publication. Information was also provided to medical practitioners and the public by recorded telephone messages and facsimile transmission. Existing quarantine protocols were modified to effect active surveillance for imported plague cases at U.S. airports. Private physicians and state and local health departments were relied on in a passive surveillance system to identify travelers with suspected plague not detected at airports. From September 27 to October 27, the surveillance system identified 13 persons with suspected plague; no case was confirmed. This coordinated response to an international health emergency may serve as a model for detecting other emerging diseases and preventing their importation.


### Surveillance for Pneumonic Plague in the United States During an International Emergency: A Model for Control of Imported Emerging Diseases

**Curtis L. Fritz, D.V.M., Ph.D., David T. Dennis, M.D., M.P.H., Margaret A. Tipple, M.D., Grant L. Campbell, M.D., Ph.D., Charles R. McCance, B.A., and Duane J. Gubler, Sc.D.**
Centers for Disease Control and Prevention, Fort Collins, Colorado, and Atlanta, Georgia, USA

In September 1994, in response to a reported epidemic of plague in India, the Centers for Disease Control and Prevention (CDC) enhanced surveillance in the United States for imported pneumonic plague. Plague information materials were rapidly developed and distributed to U.S. public health officials by electronic mail, facsimile, and expedited publication. Information was also provided to medical practitioners and the public by recorded telephone messages and facsimile transmission. Existing quarantine protocols were modified to effect active surveillance for imported plague cases at U.S. airports. Private physicians and state and local health departments were relied on in a passive surveillance system to identify travelers with suspected plague not detected at airports. From September 27 to October 27, the surveillance system identified 13 persons with suspected plague; no case was confirmed. This coordinated response to an international health emergency may serve as a model for detecting other emerging diseases and preventing their importation.

In the past 50 years, the speed of international travel, as well as the number of travelers, has accelerated, providing a mechanism for the rapid dissemination of disease agents from one country to another. For this reason, vigilant surveillance is needed to prevent the importation and spread of emergent infections. The United States needs a response plan that involves international and domestic public health officials, physicians and hospitals, and the public and can be implemented at the first indication of an international health threat.

In 1994, in response to an epidemic of pneumonic plague in India, the Centers for Disease Control and Prevention (CDC) developed and implemented an enhanced surveillance system to supplement the existing regulations concerning imported plague. The protocol described here may serve as a model for detection and control of emerging diseases imported into the United States or other countries with frequent and diverse international traffic.

### Background

In September 1994, India reported cases of plague for the first time in 28 years. Plague is caused by infection with the bacterium *Yersinia pestis*. Bubonic plague is typically acquired by the bite of fleas from infected rodents and is characterized by inguinal, axillary, and/or cervical lymphadenitis. Pneumonic plague may occur as a secondary development to the bubonic form or can be contracted by inhaling respiratory droplets from humans or other animals with plague pneumonia (1).

Bubonic plague cases were first identified by Indian health officials in the Beed District of Maharashtra State in late August. By September 24, more than 300 unconfirmed cases of pneumonic plague and 36 deaths had been reported from the city of Surat, Gujurat State, approximately 300 km west of the Beed District (2). After these reports, hundreds of thousands of Surat's two million residents fled, some to the major cities of Bombay, Calcutta, and New Delhi (3). Unconfirmed pneumonic plague cases and plague-related deaths were subsequently reported from several areas throughout India (4,5).

With the reported epidemic of plague in India, the potential for spread of the disease by infected travelers became a concern. Several countries closed their borders to Indian travelers and cargo and discontinued all flights of their air carriers to and from India (6). Because of its epidemic potential, plague is listed as a Class 1 internationally quarantinable disease in the International Health Regulations of the World Health Organization

(WHO) (7). These regulations authorize the detention and inspection of any vehicle or passenger originating in an area where a plague epidemic is in progress.

### Response to the Epidemic

CDC's domestic response to the apparent plague epidemic in India involved two simultaneous and complementary components: 1) information dissemination and education, and 2) intensified active and passive surveillance to identify and treat suspected plague patients and their contacts.

### Information Dissemination

After the initial reports from India, information on plague and the epidemic in India was urgently sought by the media, the public, medical practitioners, and public health officials throughout the United States. To meet this need, CDC circulated detailed and timely information to persons concerned with the potential plague crisis. From September 26 to 29, CDC produced six documents for distribution to public health officials and agencies: 1) a general plague outbreak notice, 2) a plague alert notice for international travelers from India, 3) a plague advisory for persons traveling to India, 4) plague treatment and prophylaxis guidelines for physicians, 5) guidelines for diagnosis and biosafety for persons handling samples from patients with suspected plague, and 6) an article on the Indian outbreak that appeared in CDC's widely circulated *Morbidity and Mortality Weekly Report (MMWR)* (8).

CDC pursued several avenues to convey information to medical practitioners and the public. Three articles on the epidemic were published in *MMWR* (September 30, October 7, and October 21) (8-10). Information on plague in general and the Indian epidemic in particular was made available on CDC's Voice Information Service, Fax Information Service, and a special plague hotline telephone number. A message intended for travelers to India concerning the perceived risks and appropriate prophylactic measures was added to the plague selections on the Voice Information Service menu. Finally, all airline passengers disembarking in the United States from India were given a plague alert notice that described the symptoms of plague and advised them to seek medical attention and notify state and federal public health authorities if they had any febrile illness within the next 7 days. The standard Health Alert Notice (yellow card) of the Division of Quarantine, CDC, was made available to all other international arriving passengers and advised them of appropriate measures in the event of illness.

### Surveillance

The second component of CDC's response was to intensify active and passive surveillance for persons entering the United States who potentially had plague. Both the active (Figure 1) and passive (Figure 2) surveillance systems identified not only persons suspected of having plague but also those who might have been exposed to a patient with plague during the contagious period.

#### *Active Surveillance System*

CDC's Division of Quarantine maintains staff at major international airports in seven U.S. cities: Honolulu, Hawaii; Seattle, Washington; San Francisco and Los Angeles, California; Chicago, Illinois; Miami, Florida; and New York, New York. At airports where the division does not have staff, officials of the Immigration and Naturalization

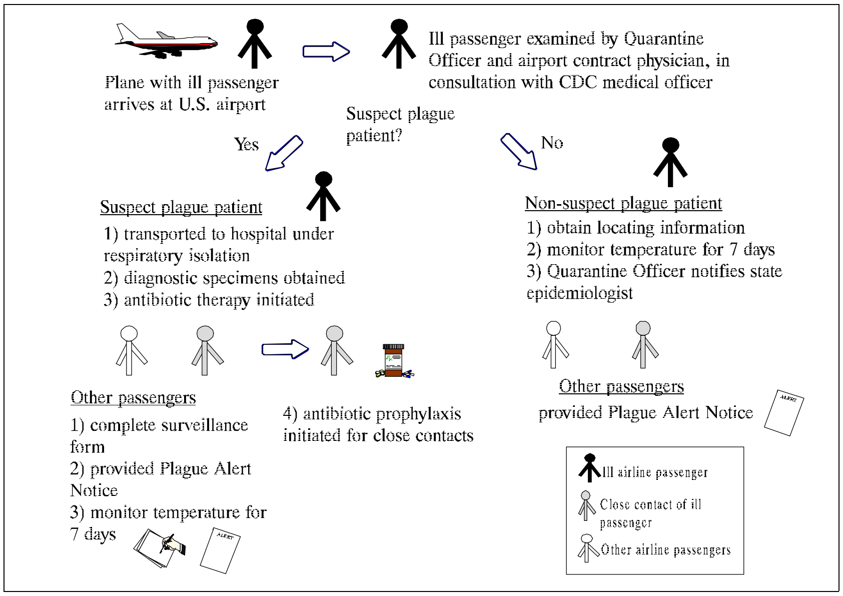

Figure 1. Active surveillance system: patient with suspected plague identified on arrival at U.S. international airport.

Service (INS), Division of Quarantine contract physicians, or both, serve as Quarantine Officers.

During the plague epidemic, crews on all commercial aircraft originating in or continuing from India were reminded of the regulations requiring them to notify the Quarantine Officer at the destination airport of any ill passengers and were instructed to be especially alert for passengers with fever, cough, or chills. When the aircraft landed, before passengers disembarked, a Quarantine Officer and a Division of Quarantine contract physician, in telephone consultation with the medical officer on call at CDC's Division of Vector-Borne Infectious Diseases, examined any passenger who reported illness and determined whether the suspicion of plague was sufficient to warrant the passenger's hospitalization and further evaluation. If deemed not likely to have plague, the passenger was placed under the surveillance of the local health department and released with instructions to consult a physician and to monitor his or her temperature for the next 7 days, the maximal incubation period for pneumonic plague after exposure (1). All other passengers were permitted to deplane and were given a copy of the plague alert notice.

If plague had not beeen ruled out as a possible cause of the passenger's illness, the passenger would have been considered a patient with suspected plague and would have been placed in isolation at the airport until he or she could be safely transported to a predetermined hospital. In the hospital, the patient would have been placed under respiratory isolation conditions, diagnostic specimens would have been obtained for testing in the CDC plague laboratory, and appropriate antibiotic treatment for plague would have been begun.

If the patient had been hospitalized, other passengers on the flight would have been informed that they were under surveillance in accordance with federal quarantine regulations. Locating information would have been obtained from all passengers, who would have been instructed to monitor their body temperature for 7 days and to report any illness to their county or state health department. Because pneumonic plague is transmitted from person to person through respiratory droplets (11) and air flow on passenger airlines is directed toward the floor (12), only passengers seated within 2 m of the patient (proximal passengers) and others with close personal contact would have been considered at reasonable risk for secondary transmission. Those proximal passengers would have been identified and advised to begin antibiotic prophylaxis and to continue it for 7 days. Had a suspected plague case been laboratory-confirmed, the state health departments and state epidemiologists would have contacted all proximal passengers and monitored completion of the antibiotic prophylaxis. All other passengers would have also been contacted to ensure that they continued to monitor themselves for febrile illness.

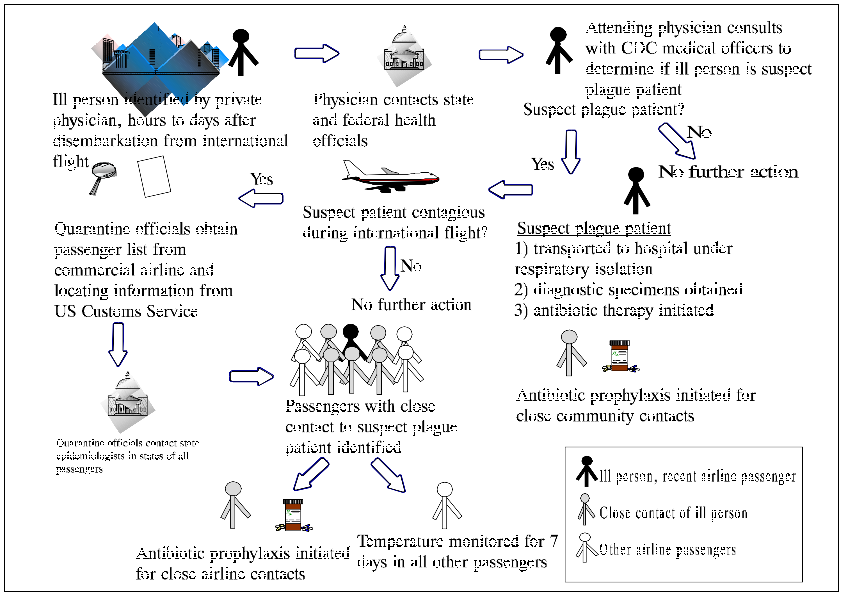

Figure 2. Passive surveillance system: patient with suspected plague identified a few hours to 7 days after arrival in the United States.

### *Passive Surveillance System*

Private physicians, hospitals, and local public health officials were relied on to identify international air travelers from India who became ill within a short period (from hours to 7 days) after disembarkation and report the illness to the appropriate state and federal public health officials. The attending physician, in consultation with the CDC medical officer on call, then determined on the basis of clinical and epidemiologic evidence whether the ill person had a reasonable likelihood of having plague. If so, the patient would have been placed under respiratory isolation in a hospital, diagnostic specimens would have been obtained, and antibiotic treatment would have been initiated. Close contacts of the suspected plague patient during the putative contagious period would have been identified and advised to begin antibiotic prophylaxis.

A concerted effort would have been made to determine the time the suspected plague patient became symptomatic, and thereby contagious (13), relative to the person's arrival in the United States. If the patient had been symptomatic at the time of the flight, a passenger list would have been obtained from the airline and the U. S. Customs Service. State epidemiologists in the states of residence of all passengers would have been informed of the need to contact and maintain surveillance of passengers within their jurisdiction who were possibly secondarily exposed. If seating assignments for the flight could be obtained, passengers seated within 2 m of the patient would have been advised to begin antibiotic prophylaxis; all other passengers would have been instructed to monitor their temperature for 7 days and to report any illness to state health officials.

### Results

On September 29, plague information documents were sent by electronic mail or fax to four Executive Committee members and 50 members of the Council of State and Territorial Epidemiologists, 60 members of the Association of State and Territorial Public Health Laboratory Directors, 40 Executive Board members and 50 state representatives of the National Association of County and City Health Officials, 132 officers in CDC's Epidemic Intelligence Service (EIS), 15 field supervisors of CDC's Field Epidemiology Training Program, and one representative each in the U. S. Department of State and the Quarantine Health Services in Canada. Although an exact count is not available, more than 3,000 persons probably received these documents directly from CDC or secondarily through other agencies.

From September 27 to October 31, the CDC Voice Information Service received 6,665 calls accessing information about plague; 2,692 of these calls were received through the special plague hotline number. During this same period, 5,589 documents about plague were requested and sent by the CDC Fax Information Service.

On October 25, 1994, after an on-site investigation in India, a WHO team of scientists that included four CDC staff members, determined that the plague epidemic was of more limited scope than previously believed, and recommended the lifting of travel restrictions. On October 27, 1994, CDC authorized a stand-down of the heightened surveillance system at all ports of entry and a return to normal operations. During the 30 days that the surveillance system was in place, 13 airline travelers arriving in the United States were evaluated. Six patients with suspected plague were identified and evaluated in airports—JFK and La Guardia in New York City (four), Dallas-Fort Worth (one), Chicago-O'Hare (one)—and seven by private physicians in New York City (five), Albany, New York (one), and St Louis, Missouri (one). All 13 had a history of recent travel in India. None was found to have plague. Symptoms of illness included fever (eight), cough (six), vomiting (four), and malaise (three). The final diagnoses of persons evaluated were viral syndrome (four), malaria (two), concurrent malaria and dengue (one), typhoid (one), end-stage liver failure (one), and no illness (one) (14). The final diagnosis was unspecified in three patients.

### Discussion

Plague pandemics have occurred throughout history (15). In the European epidemic known as Black Death, from 1345 to 1360, an estimated quarter of the world's known population of 24 million died. Originating in central Asia and carried by ship to Sicily, the disease spread east to China, south to Africa, north to Russia and Scandinavia, and west to Greenland in only a few years. Plague in North America can be traced historically to infected rats aboard ships from the Far East that docked in California during the early 20th century (16). Today, air travel that can transport a person anywhere in the world within 24 hours expands the opportunity for rapid spread of a transmissible disease like pneumonic plague. The potential for pneumonic plague to spread by air travel to the United States during the recent Indian epidemic elicited considerable public concern (7).

Although rare, plague is enzootic in the United States, and 10 to 15 human cases are reported each year; typically only one or two of these are pneumonic plague cases (17). Thus, most public health officials and medical practitioners in this country have limited experience with plague (18). When the Indian epidemic began, detailed and reliable information from India was sparse; therefore, CDC disseminated factual and comprehensive information regarding pneumonic plague and the Indian epidemic to public health officials, physicians, and private citizens. The development and distribution of the e-mail, voice, fax, and printed documents were coordinated through a single branch within CDC, which ensured the accuracy and timeliness of the information conveyed. By serving as the central clearing house for international and domestic reports, CDC was able to gather and redistribute information rapidly and efficiently. Updates in *MMWR* contained data obtained within hours of publication. Most public health officials and agencies were accessible immediately by electronic mail or fax, and group mailing codes were constructed to facilitate simultaneous communication. These timely updates of information, which included periodic results of the enhanced surveillance system, heightened awareness of the public health threat and encouraged participation by health practitioners in the passive component of the surveillance system.

Like the information network, the surveillance system was centrally coordinated at CDC, but it relied on the contributions of many agencies and individuals to function effectively. Federal, state, and local health officials, the Immigration and Naturalization Service, the U.S. Customs Service, commercial businesses (passenger airlines), medical practitioners, hospital personnel, and the public played key roles in the successful implementation of the system. Many state and local health departments made additional efforts to alert the medical community to the potential for imported plague cases, to reiterate the surveillance protocol, and to emphasize the importance of obtaining a travel history from any patient with unexplained fever (14). This distribution of responsibility through the established public health network was essential to effective surveillance.

In 1992, the Institute of Medicine's Committee on Emerging Microbial Threats to Health recommended that surveillance of international infectious diseases be implemented and coordinated by a single government agency, ideally CDC (19); subsequently, CDC developed a comprehensive strategy for preventing emerging infectious diseases in the United States (20). In its response to the Indian plague epidemic, rather than constructing a new system specific to this emergency, CDC used a surveillance protocol that built on the existing quarantine framework to utilize trained staff in a position to readily respond. Future responses to the threat of importation of communicable diseases with epidemic potential will require a similar network of individuals and agencies, with specific roles and responsibilities but sufficiently flexible to adapt to the particular epidemiologic circumstances. A system similar to the one described here was put in place in response to the Ebola outbreak in Zaire in April and May 1995 (21).

A surveillance system must be effective without becoming overly burdensome to either those conducting the surveillance or those under surveillance; it must safeguard the public health without inhibiting commerce or interfering with individual freedoms. In the 1370s, during the latter years of Black Death, nautical travelers to the Republic of Ragusa, now part of Italy, were detained for 40 days (from which the word "quarantine" [*quaranti giorni*] derives) (15), a detention period inappropriately long in light of the current knowledge of plague's incubation period of 2 to 7 days (1). In the recent outbreak, closure of airports to all flights from India, compulsory quarantine of all international travelers, and an embargo of trade with India were extreme measures given the epidemiology of plague and the risk of importing a case (18). Primary surveillance efforts were focused at critical control points, i.e., international airports, where personnel resources for identification and control of imported plague cases are maximally efficient. The secondary system, utilizing private physicians and state and local health departments, permitted continued surveillance that was less intensive, but geographically expansive, without placing an unnecessary burden on international air travelers.

If a case of plague had been confirmed in an airline passenger, tracing passengers at risk would have been a substantial undertaking. Depending on the interval between disembarkation and diagnosis, hundreds of persons might have had to be located across the country. In addition to 39 of CDC's Epidemic Intelligence Service (EIS) Officers stationed in state and local health departments, 10 EIS Officers in CDC centers in Atlanta, Georgia, Cincinnati, Ohio, Washington D.C., and Fort Collins, Colorado, were recruited to assist state and local health departments in tracing contacts if necessary. EIS Officers have often been called to assist in public health crises in which a large complement of epidemiologists was required; in 1993, 13 EIS Officers were among the scientists and public health officials assembled during the outbreak of hantavirus pulmonary syndrome in the southwestern United States (22). Because a rapid response to importation of a disease with epidemic potential often requires a national team of epidemiologists to assist local public health agencies, the Institute of Medicine and others have recommended the expansion and continued support of CDC's EIS program (19,20,23).

The surveillance system's first line of detection for plague cases depended on airline personnel, Immigration and Naturalization Service and U.S. Customs officials for the active component, and private physicians and health care providers for the passive component. Since the former are not trained medical personnel and may not detect an ill traveler in the absence of obvious signs and symptoms, and the latter may not be sufficiently alerted to the possibility of plague, diagnosis of some plague cases could have been delayed and not been efficiently detected by the surveillance system. It is unrealistic to expect any system to effectively screen all travelers returning from areas of recognized disease outbreaks. It is impossible to assess the sensitivity of the described surveillance system since no cases of pneumonic plague were identified either within or outside the system. In retrospect, the risk for an imported plague case was quite small, since the epidemic in India was limited in time and space and had far fewer cases than originally suspected (24). The WHO investigative team found no evidence of transmission in metropolitan areas other than Surat. Most of the patients with suspected plague in Surat came from poor neighborhoods, residents of which would be unlikely to travel internationally. In addition, the short incubation period and severe symptoms of pneumonic plague and the rapid deterioration of the patient's condition, substantially limited the contagious period and the opportunity for secondary transmission.

Although the epidemic potential for plague makes it a good model for developing emerging disease response capabilities, the direct applicability of this program for other emerging diseases may not be straightforward. The above protocol was developed in response to a regionally limited outbreak that occurred during a relatively brief period, similar to the recent Ebola outbreak in Zaire (21). To detect emerging diseases in the absence of a recognized outbreak, surveillance would need to be maintained at some baseline level for an indefinite period. Compliance with the enhanced plague surveillance protocol during the short period it was in effect appears to have been excellent, but how compliance might have waned over weeks to months is unknown. In addition, the protocol was specific to plague, a well-characterized disease with well-described pathogenesis and clinical features. The severe manifestations of pneumonic plague, the short incubation and contagion periods, and the availability of reliable diagnostic tests allowed for a focused protocol that could confidently identify cases. Other emerging diseases may be less well characterized, or even entirely unknown, and may require surveillance protocols of lesser specificity. Nevertheless, the plague surveillance system was broad enough (and consistent with the Institute of Medicine's recommendation that a global infectious disease surveillance system implement broad reporting criteria for detection of emerging diseases [19]) to identify four persons who had other potentially fatal notifiable infectious diseases.

### Acknowledgments

The authors thank Dr. May C. Chu, Dr. Robert B. Craven, Mr. Thomas A. DeMarcus, Ms. Rosamond R. Dewart, Dr. Kenneth L. Gage, Dr. Kathleen A. Orloski, Mr. Tony D. Perez, Dr. Jack D. Poland, Dr. Martin E. Schriefer, Mr. Thomas W. Skinner, Dr. Ofelia C. Tablan, and Dr. Theodore F. Tsai, (CDC), and Dr. Brian Gushulak, (Health Canada), for expert consultation and direct assistance; Ms. Mary Ellen Fernandez and Ms. Edwarda O. Lee, (CDC), for administrative support; Ms. Kathy A. Bruce, Ms. Rebecca L. Deavours, Ms. Anna M. Jimenez, and Ms. Karen A. Peterson (CDC), for secretarial support; Mr. Jerome R. Cordts (Association of State and Territorial Public Health Laboratory Directors), Mr. David E. Custer and Ms. Nancy Rawding, (National Association of County and City Health Officials), Mr. Willis R. Forrester and Ms. Kathy F. Getz (Council of State and Territorial Epidemiologists), Dr. Martin Wolfe, (U.S. Department of State), and Ms. Patsy R. Bellamy, Ms. Pamela K. Eberhardt, and Mr. Clyde S. Furney, Jr. (CDC), for assistance with rapid dissemination of information; Mr. Richard Heffernan, and Dr. Marcelle Layton, (New York City Department of Health), and Dr. Rosalind J. Carter (New York City Department of Health and CDC), for assistance in conducting surveillance.

### References

1. Benenson AS, editor. Control of Communicable Diseases in Man, 15th ed. Washington, DC: American Public Health Association, 1990.
2. Desai D, Samuel I. Surat flounders against medical crisis. The Indian Express 24 September 1994.
3. Gupta S. 600,000 have fled Surat. The Statesman 25 September 1994.
4. World Health Organization, Regional Office for Europe. Plague in India. Communicable Disease Report (CD News) Issue 4; 3 October 1994.
5. Friese K, Mahurka U, Rattanani L, Kattyar A, Rai S. The plague peril. Are you at risk? India Today 15 October 1994.
6. Post T, Clifton T, Mazumdar S, Cowley G, Raghavan S. The plague of panic. Newsweek 10 October 1994:40-1.
7. World Health Organization. International Health Regulations. 3rd ed. Geneva, Switzerland: WHO, 1983:26-9.
8. Centers for Disease Control and Prevention. Human plague—India, 1994. MMWR 1994;43:689-91.
9. Centers for Disease Control and Prevention. Update: Human plague—India, 1994. MMWR 1994;43:722-3.
10. Centers for Disease Control and Prevention. Update: Human plague—India, 1994. MMWR 1994;43:761-2.
11. Craven RB. Plague. In: Hoeprich PD, Jorday MC, Ronald AR, editors. Infectious diseases: a treatise of infectious processes. 5th ed. Philadelphia: Lippincott, 1994:1302-12.
12. National Research Council. The airliner cabin environment: air quality and safety. Washington, DC: National Academy Press; 1986.
13. Poland JD, Barnes AM. Plague. In: Steele JH, editor. CRC handbook series in zoonoses: section A: bacterial, rickettsial, and mycotic diseases. Boca Raton, FL: CRC Press; 1979:515-58.
14. Centers for Disease Control and Prevention. Detection of notifiable diseases through surveillance for imported plague—New York, September-October 1994. MMWR 1994;44:805-7.
15. Cartwright FF. Disease and history. New York: Dorset Press, 1972.
16. Craven RB, Barnes AM. Plague and tularemia. Infect Dis Clin North Am 1991;5:165-75.
17. Centers for Disease Control and Prevention. Summary of notifiable diseases, United States. 1993. MMWR 1993;42:45.
18. Campbell GL, Hughes JM. Plague in India: a new warning from an old nemesis. Ann Intern Med 1995;122:151-3.
19. Lederberg J, Shope RE, Oaks SC, editors. Emerging infections: microbial threats to health in the United States. Washington, DC: National Academy Press, 1992.
20. Centers for Disease Control and Prevention. Addressing emerging infectious disease threats: a prevention strategy for the United States. Atlanta, GA: U.S. Department of Health and Human Services, Public Health Service, 1994.
21. Centers for Disease Control and Prevention. Outbreak of Ebola viral hemorrhagic fever—Zaire, 1995. MMWR 1995;44:381-2.
22. Centers for Disease Control and Prevention. Update: outbreak of hantavirus infection—southwestern United States, 1993. MMWR 1993;42:441-3.
23. Henderson DA. Surveillance systems and intergovernmental cooperation. In: Morse SS, editor. Emerging viruses. New York: Oxford University Press, 1993:283-9.
24. World Health Organization, South-East Asia Regional Office. Plague in India: World Health Organization team executive report. Geneva, Switzerland: WHO, December 1994.
